# Structural imaging findings on non-enhanced computed tomography are severely underreported in the primary care diagnostic work-up of subjective cognitive decline

**DOI:** 10.1007/s00234-019-02156-6

**Published:** 2019-01-17

**Authors:** Claes Håkansson, Gustav Torisson, Elisabet Londos, Oskar Hansson, Danielle van Westen

**Affiliations:** 1 Diagnostic Radiology, Institution for Clinical Sciences, Lund University, Lund, Sweden; 2 Image and Function, Skåne University Hospital, Lund, Sweden; 3 Department of Translational Medicine, Clinical Infection Medicine, Lund University, Malmö, Sweden; 4 Department of Clinical Sciences, Clinical Memory Research Unit, Lund University, Lund, Sweden; 5 Memory Clinic, Skåne University Hospital, Malmö, Sweden

**Keywords:** Dementia, Visual rating scales, Computed tomography

## Abstract

**Purpose:**

The purpose of this study was to investigate how structural imaging findings of medial temporal lobe atrophy (MTA), posterior cortical atrophy (PCA), global cortical atrophy (GCA), white matter changes (WMC), and Evans’ index/width of lateral ventricles (EI/WLV) are reported in the primary care diagnostic work-up of patients with subjective cognitive decline or mild cognitive impairment.

**Methods:**

We included 197 patients referred to a non-enhanced computed tomography (NECT) as part of the diagnostic work-up. We compared the frequencies of reported findings in radiology reports written by neuroradiologists and general radiologists with actual pathological findings in a second view done by a single neuroradiologist using the MTA, PCA, GCA, WMC, and EI/WLV visual rating scales. Structural findings were also compared to cognitive tests.

**Results:**

We found that MTA and PCA were clearly underreported by both neuroradiologists and general radiologists. The presence of GCA and WMC was also underreported among general radiologists. Only MTA showed a clear association with cognitive test results.

**Conclusions:**

We believe that the use of visual rating scales should be put into clinical practice to increase the yield of clinical NECT exams in the investigation of cognitive impairment. Special emphasis should be put on reporting MTA.

## Introduction

Dementia is a clinical condition caused by a spectrum of diseases where Alzheimer’s disease (AD) is the most common among elderly [1, 2]. Alzheimer’s disease has an insidious onset with progressive memory impairment and a prodromal phase starting decades before clinical onset, a phase often denoted mild cognitive impairment (MCI). Patients with MCI have an increased risk of later developing AD but early stages of AD and MCI can be difficult to separate [3]. The incidence of dementia is predicted to increase in the next 20 to 30 years and validation of new therapies requires early identification of patients. Treatment does not prevent the disease but preserves autonomy and independence for the patients for a longer time thereby reducing the health care costs and increasing the quality of life [2, 4].

The initial diagnostic work-up includes basic assessments of cognitive domains and structural neuroimaging. Structural neuroimaging, i.e., computed tomography (CT) or magnetic resonance imaging (MRI), is used to exclude potentially treatable causes of dementia but also identifying structural findings that may support the suspected diagnosis [1, 5, 6]. As an example, medial temporal lobe atrophy (MTA) is the imaging hallmark of AD while the absence of MTA and the presence of severe white matter changes (WMC) may lead to the suspicion of vascular dementia. Certain atrophy patterns can be identified by applying different visual rating scales. A number of well-validated visual rating scales are available to grade patterns of atrophy such as MTA, posterior cortical atrophy (PCA), global cortical atrophy (GCA), and WMC [7, 8]. Most scales are developed for MRI but visual assessment scales for MTA, GCA, and WMC have been tried for CT in a few studies [9–11].

We want to investigate if pathological structural findings are underreported in radiology reports during the initial diagnostic work-up initiated by primary care general physicians. We also want to study whether or not there is a difference between neuroradiologists and general radiologist in the reporting of structural findings. The structural findings will be compared to clinical assessments of cognition to assess the clinical relevance.

## Materials and methods

### Material

This study is a retrospective assessment of clinical routine non-enhanced CT (NECT) performed as part of an initial diagnostic work-up in a group of patients with anamnestic subjective cognitive decline (SCD). Patients were retrospectively recruited from a cohort in the prospective Swedish Biomarkers For Identifying Neurodegenerative Disorders Early and Reliably (BioFINDER) study. The eligible patient had performed a NECT within 12 months prior to the BioFINDER study baseline.

All clinical NECT exams were performed according to local routine brain scan protocols. All images had been reformatted in 5-mm slices in the axial plane (parallel to the orbitomeatal plane), 3-mm slices in the coronal plane (parallel to the axis of the brain stem), and 3-mm slices in the sagittal plane. The original data acquisitions (transverse 0.75-mm slices) were available to perform these reconstructions in the picture archiving and communication system (PACS). Data on image acquisition was retrospectively retrieved from the Digital Imaging and Communications in Medicine (DICOM) head in our PACS. Exams were performed in helical scan mode with pixel spacing of 0.43 × 0.43 and *z*-axis automatic exposure control was used. Further data on image acquisition are presented in Table 1. All images were viewed in our PACS on Sectra® Workstation IDS7® 19.1 (Sectra AB) with a center windowing of 40 HU and a width windowing of 80 HU. The referral forms and dates of clinical examinations were simultaneously made available in our regional PACS.

**Table 1:** Data on image acquisition and number of patients examined in each scanner model (*N* = 197)

Manufacturer	Philips				Siemens SOMATOM			Toshiba
	Brilliance 64®	Ingenuity®	Mx8000®	iCT 128®	Sensation 16®	Definition AS®	Definition Flash®	Aquilion ONE®
Patients (*n*)	98	13	7	1	45	9	17	7
mAS	350	320	300	410	320	310	300	300
kV	120	120	120	120	120	120	120	120
Collimation	0.63	0.63	NA*	NA*	0.6	0.6	0.6	NA*
Pitch factor	0.39	0.52	NA*	NA*	0.55	0.55	0.55	0.65

Note: Data retrospectively retrieved from the Digital Imaging and Communications in Medicine (DICOM) head and scan protocols available in PACS

*No available data (*n* = 15)

### Rating of structural findings

Visual rating of MTA was done on images in the coronal plane according to Scheltens’ 5-point MTA scale (from 0: no atrophy to 4: severe atrophy) [12, 13]. The scores were dichotomized into normal (0–1 if age < 75 years, 0–2 if ages > 75 years) and pathological (MTA 2–4 if age < 75 years, 3–4 if age > 75 years) [13–16]. Visual rating of PCA was done on coronal, sagittal, and axial images using Koedam’s 4-point PCA-scale (from 0: no atrophy to 3: severe atrophy). A cut-off score of ≥ 1 independent of age has been suggested but mild changes may be present in elderly and a cut-off score of ≥ 2 has been shown to have a better specificity [15, 17, 18]. Consequently, scores were dichotomized into normal (PCA 0–1 regardless of age) and pathological (PCA 2–3 regardless of age). For both MTA and PCA, the left and right sides were assessed separately and in the case of different scores between sides, the highest score was used.

A clinically applicable version of Pasquier’s 4-point GCA scale was used to assess the overall loss of cortical volume (from 0: no atrophy to 3: severe atrophy) [8, 19]. A cut-off score of ≥ 1 has been suggested regardless of age but GCA increases with age and a score of 1 can also be seen in normal aging [15, 18]. The scores were dichotomized into normal (GCA 0–1 regardless of age) and pathological (GCA 2–3 regardless of age). We used Fazekas’ 4-point WMC scale to assess WMC (from 0: no or a single lesion to 3: confluent lesions) [20]. The scores were dichotomized into normal (WMC 0 if age < 65 years and 0–1 if age > 65 years) and pathological (WMC 1–3 if age < 65 years and 2–3 if age > 65 years) [8]. The assessments were done on axial slices.

Evans’ index (EI) is the ratio between the width of the anterior horns of the lateral ventricles and the widest inner diameter of the skull [21]. It gives an estimation of the size of the lateral ventricles and indirect estimation of central atrophy. Evans’ index was originally based on encephalographic measurements but has been modified for axial CT. New cut-offs corrected for age and gender have been proposed as follows (age, males/females): 65–69 years 0.34/0.32, 70–74 years 0.36/0.33, 75–79 years 0.37/0.34, and 80–84 years 0.37/0.36 [22]. For patients < 65 years EI ≥ 0.30 was considered pathological regardless of gender. The measurements were done on axial slices using tools available in our PACS and dichotomized into normal and pathological accordingly.

### Review of clinical reports

All clinical reports were re-evaluated and all clinical NECT were re-rated in a second view done by a board-certified neuroradiologist (C.H) with 5-year experience in general radiology and additional 4-year experience in neuroradiology. The abovementioned visual rating scales were used and the rater had access to online templates for the different scales (available at www.radiologyassistant.com).

The radiology reports were re-evaluated with respect to quantitative (e.g., “mild,” “moderate,” or “severe”) or qualitative description of MTA (e.g., “enlarged temporal horns”), PCA (e.g., “widened parietal sulci”), GCA (e.g., “age-related widening of sulci”), WMC (e.g., “chronic white matter changes”), and EI/WLV (e.g., “enlarged lateral ventricles”). The described findings in the radiology reports were quantitively re-scored in a 4-point scale adapted from Torisson et al. as follows: NA: Finding not mentioned, 0: Described as normal, 1: Described as mild or mentioned but not quantified, 2: Findings described as moderate, and 3: Findings described as severe. For EI/WLV, a score of 1–3 was considered pathological regardless of age and gender. For the remaining pathologies, a score of 0–1 was considered normal and a score of 2–3 was considered pathological regardless of age and gender [11]. The reported findings from the radiology reports were compared to findings from the second view.

A subgroup analysis comparing radiology reports from board certified neuroradiologist and general radiologists was also performed. In our country board, certified general radiologists have at least 5 years of training in general radiology; this includes at least 6 months in neuroradiology, and board-certified neuroradiologists have an additional 2 years training in neuroradiology. Senior general radiologists and neuroradiologists have at least an additional 3 years of clinical experience within their field. We defined a neuroradiologist as a person having held a position at our neuroradiology department for at least 2 years or declaring to be a board-certified neuroradiologist. A report was considered issued by a neuroradiologist if the final approval was issued by a neuroradiologist. For the assessment of cognition data on education level, the mini-mental state exam (MMSE), the clock drawing test (CDT), and the Alzheimer’s disease assessment scale (ADAS) ten-word recall were retrieved from the baseline data. The MMSE measures orientation, registration, attention and calculation, recall, and language. An overall score between 0 and 30 is given and the lower the score the greater the level of cognitive impairment [23]. In the CDT, patients are instructed to draw an analogue clock and a score from 0 to 5 is given and a score of 0–3 is considered abnormal [24]. The ADAS ten-word recall measures the anterograde memory functions and is scored between 0 and 10, the higher the score the poorer the result [25].

### Statistics

The association between cognitive data and the dichotomized rating was calculated using the Mann-Whitney *U* test. The differences in reported pathologies between radiology reports and the second view were calculated using McNemar’s test for related samples. For estimation of intra-rater agreement on dichotomized rating, an additional review was performed by the same rater (C.H) on all patients (*N* = 197) after 3 months and Cohen’s kappa was calculated. The level of agreement was defined according to Landis and Koch [26]. All data were prepared in Microsoft® Excel (Microsoft Corporation) and all calculations were done using IBM® SPSS® Statistics version 24 (IBM Corporation). A *p* < .05 was considered statistically significant and after the Bonferroni correction, a *p* ≤ .002 was considered significant.

## Results

We retrospectively identified 250 eligible patients who had performed a NECT 12 months prior to the BioFINDER imaging baseline. Of these, 43 had not performed NECT as part of a memory impairment diagnostic work-up and referrals were missing for 10 patients leaving 197 patients who were included in the study. The included patients had performed a NECT within 12 months (median 146 days, interquartile range 117.5 days) from baseline. Cognitive impairment was mentioned in 193 (98.0%) of the 197 referrals and personality changes were mentioned in ten (5.1%). Ninety-six of the 197 (48.7%) patients were examined at our department at Skånes universitetssjukhus, a tertiary academic hospital in southern Sweden with a dedicated department in neuroradiology, and 101 (51.3%) patients were examined at local secondary-level hospitals with no dedicated departments in neuroradiology. In total, 182 of the 197 (92.4%) referrals were issued by general practitioners in the primary care which is the standard procedure in the initial diagnostic work-up in our country. All cognitive data were retrieved within 12 months from NECT (median 84 days, interquartile range 82.5 days). None of the patients were diagnosed with dementia at baseline.

The clinical NECT exams had in total been viewed by 50 different general radiologists and 30 different neuroradiologists. Thirty-two of the 50 general radiologists and nine of the 30 neuroradiologists had issued one report each. One of the neuroradiologists had issued eleven reports and two had issued eight reports. One of the general radiologists had issued five reports and five had issued four reports each. All of the latter were senior neuroradiologists or radiologists. At our department, 87 out of 96 (90.6%) reports were issued by neuroradiologists and at the secondary-level clinics, 25 out of 101 (24.8%) reports were issued by neuroradiologists. Evans’ index was not mentioned in the radiology reports at all. For the sake of comparison, EI and WLV were therefore grouped together (EI/WLV). (For the display of further baseline data, see Table 2.)

**Table 2:** Baseline data of study population (*N* = 197)

Characteristic	
Age (years)	71.4 (8.1)
Female (%)	46.2
Reports issued by neuroradiologists (*n*)	112
Reports issued by general radiologists (*n*)	85
Education level (years)	12.0 (5)
MMSE (score)	28.0 (3)
CDT (score)	5.0 (2)
ADAS (score)	5.0 (4)

Note*:* All data presented as median (interquartile range) where applicable. *MMSE* mini-mental state examination, *CDT* clock drawing test, *ADAS* Alzheimer’s diseases assessment scale ten-word recall

**Fig. 1:**
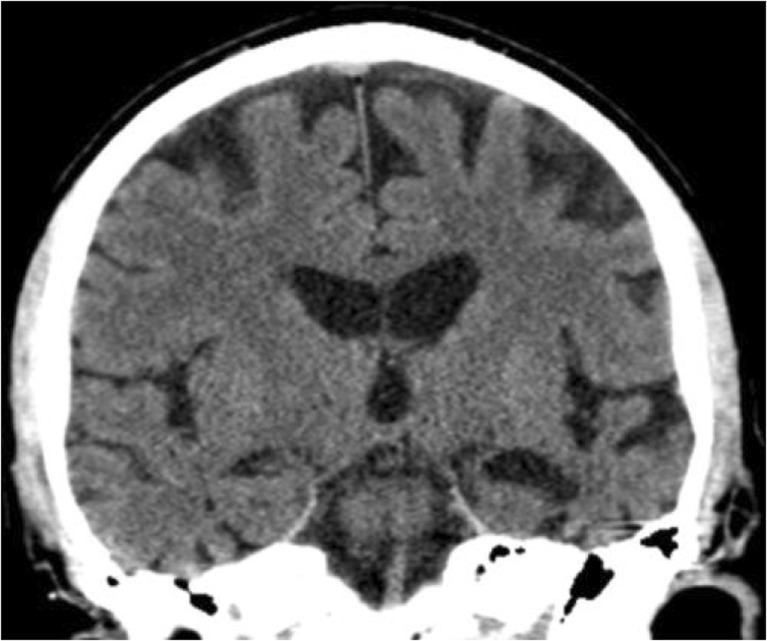
Sixty-three-year-old male where MTA was mentioned but not quantified in the original report. Right side rated as normal, MTA 1, and left side rated as pathological, MTA 4, in the second reading

**Fig. 2:**
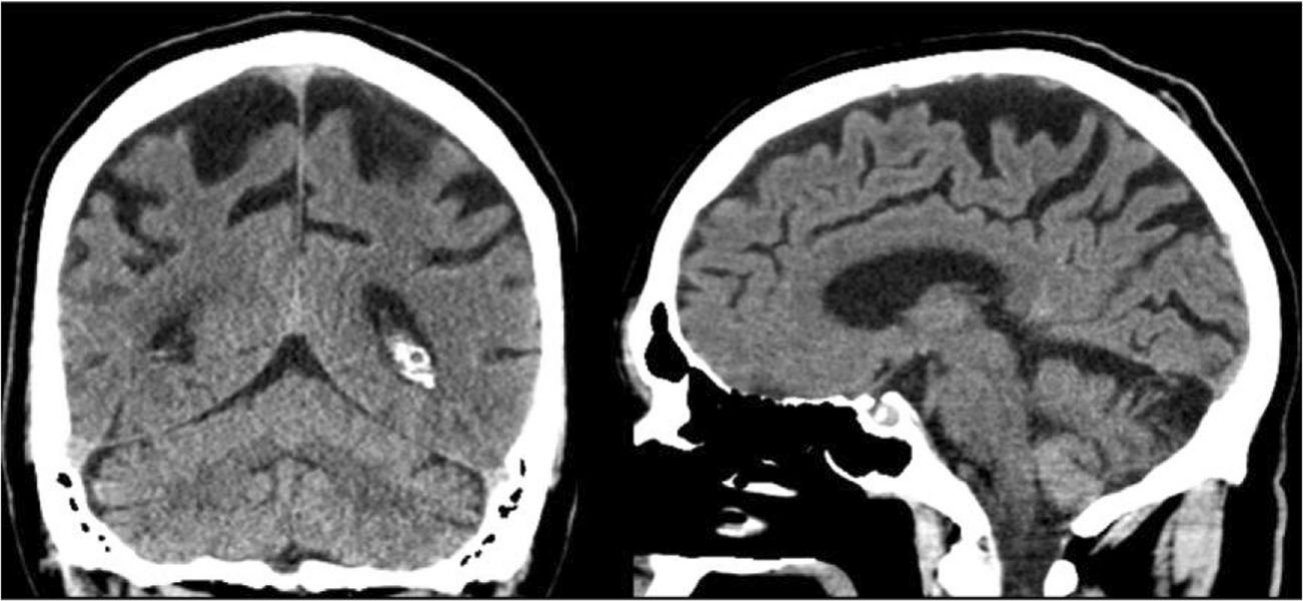
Sixty-eight-year-old male where sulci were reported as normal in the original report. Rated as pathological, PCA 2, in the second reading

When total frequencies of reported pathological findings in the reports were compared to pathological findings in the second view, there was a significant underreporting of all pathologies, except for pathological EI/WLV. (See Table 3 for detailed data.) In a sub-analysis, reports issued by neuroradiologists and general radiologists were compared in a similar manner. Among neuroradiologists, there was a significant underreporting of pathological MTA and PCA but not of the other pathologies. (See Table 4 for detailed data.) Among general radiologists, there was a significant underreporting of all pathologies except for EI/WLV. (See Table 5 for detailed data.) An image example where pathological MTA was not quantified in the original report is given in Fig. 1 and an image example where PCA was not reported is given in Fig. 2.

**Table 3:** Frequencies of findings reported as pathologic by neuroradiologists and general radiologists compared to findings judged as pathologic in the second view (*N* = 197)

Pathology	Clinical reports (%)		Second view (%)	*p*
	Not mentioned	Pathologic	Pathologic	
MTA	70.1	2.0	19.3	< .001
PCA	92.9	1.5	11.2	< .001
WMC	25.9	13.2	22.3	.001
GCA	34.0	3.0	16.2	< .001
EI/WLV	33.0	13.2	14.2	.860

Note: McNemar’s test; *p* < 0.05 represents significant underreporting. All data presented as percentages of pathological findings in radiology reports and on second view

**Table 4:** Frequencies of reported pathologies compared to findings judged as pathologic in the second view: neuroradiologists (*n* = 112)

Pathology	Reported as pathologic (%)		*p*
	Clinical reports	Second view	
MTA	2.7	17.0	< .001
PCA	0.9	6.3	.031
WMC	20.5	27.7	.077
GCA	4.5	10.7	.065
EI/WLV	11.6	15.2	.629

Note: McNemar’s test; *p* < .05 represents significant underreporting

**Table 5:** Frequencies of reported pathologies compared to findings judged as pathologic in the second view: general radiologists (*n* = 85)

Pathology	Reported as pathological (%)		*p*
	Clinical reports	Second view	
MTA	1.2	22.4	< .001
PCA	2.4	17.6	.001
WMC	3.5	15.3	.002
GCA	1.2	23.5	< .001
EI/WLV	15.3	14.1	1.000

Note: McNemar’s test; *p* < .05 represents significant underreporting

Cohen’s kappa was performed to determine intra-observer agreement for dichotomized rating in an additional review of all NECT exams (*N* = 197). There was strong agreement for pathological EI/WLV (*κ* = 0.911 (95% CI, 0.823 to 0.999), *p* < .001), pathological MTA (*κ* = 0.902 (95% CI, 0.824 to 0.980), *p* < .001), and pathological PCA (*κ* = 0.817 (95% CI, 0.683 to 0.951), *p* < .001). There was substantial agreement for pathological WMC (*κ* = 0.795 (95% CI, 0.687 to 0.903), *p* < .001) and pathological GCA (*κ* = 0.662 (95% CI, 0.510 to 0.814), *p* < .001).

A Mann-Whitney *U* test was performed to analyze the association between pathological findings and cognition. There was no association between education level and any of the five pathologies (data not shown). There was a very strong association between pathological MTA and ADAS scores and pathological MTA and CDT scores. There was a strong association between pathological MTA and MMSE scores. There was a strong association between pathological PCA and ADAS score and a very strong association between pathological PCA and age. There was a strong association between pathological WMC and CDT. Pathological GCA had a strong association with age and a weak association with pathological ADAS. Pathological EI/WLV was associated only with age. (See Table 6 for detailed data.) The association between pathological MTA and CDT and pathological MTA and ADAS was the only to sustain a Bonferroni correction.

**Table 6:** Association between each of the five pathologies and cognitive domains (*N* = 197)^a^

	Pathologic	Normal	*p* value
MTA			
Total (*n*)	38	159	
Age (years)	72.8 (5.6)	70.3 (8.5)	.197
MMSE	27.0 (3.0)	28.0 (3.0)	.004
ADAS	7.0 (3.0)	5.0 (4.0)	.001*
CDT	4.0 (3.0)	5.0 (1.0)	.001*
WMC			
Total (*n*)	44	153	
Age (years)	72.6 (7.6)	70.9 (7.8)	.414
MMSE	28.0 (3.0)	28.0 (3.0)	.805
ADAS	5.5 (3.0)	5.0 (4.0)	.205
CDT	4.0 (2.0)	5.0 (1.0)	.015
EI/WLV			
Total (*n*)	28	169	
Age (years)	68.6 (9.3)	72.0 (7.7)	.023
MMSE	28.0 (3.8)	28.0 (3.0)	.394
ADAS	6.0 (4.8)	5.0 (4.0)	.316
CDT	4.5 (2.0)	5.0 (1.0)	.150
PCA			
Total (*n*)	22	175	
Age (years)	75.1 (9.1)	70.7 (8.0)	.007
MMSE	28.0 (1.5)	28.0 (3.0)	.631
ADAS	7.0 (3.3)	5.0 (4.0)	.021
CDT	5.0 (2.0)	5.0 (1.0)	.667
GCA			
Total (*n*)	32	165	
Age (years)	74.2 (9.6)	70.7 (7.9)	.008
MMSE	28.0 (2.8)	28.0 (3.0)	.428
ADAS	6.5 (4.0)	5.0 (4.0)	.045
CDT	4.0 (2.0)	5.0 (1.0)	.090

Note: The Mann-Whitney *U* test; *p* < .05 represents a significant association. All data presented as median (interquartile range) where applicable. *MMSE* mini-mental state examination, *CDT* clock drawing test, *ADAS* Alzheimer’s diseases assessment scale ten-word recall

*Significant after the Bonferroni correction, *p* ≤ .05/25 ≤ .002

^a^There was no significant association between education level and any of the five pathologies why these data are not shown

Apart from the abovementioned findings, 23 patients also had other pathologies. Of these, eight had a lacunar (defined as < 15 mm in size) infarct, six had a large (defined as > 15 mm in size) infarct, three had an arachnoid cyst, one had basal ganglia calcifications, one had a remaining cavum septum pellucidum, one had a pineal cyst, and one had a small meningioma. Of the remaining, one patient had both a large infarct and basal ganglia calcifications and another had both a large infarct and a lacunar infarct.

## Discussion

In this study, pathological MTA, PCA, GCA, and WMC were found to be severely underreported in the primary diagnostic work of patients with SCD undergoing a NECT. A subgroup analysis showed that among general radiologists, all findings except EI/WLV were underreported and among neuroradiologists, MTA and PCA were underreported.

The first aim of this study was to investigate if structural findings associated with dementia were underreported in clinical reports. Radiologists tend to differ in their reporting styles depending on local traditions, personal preferences, and experiences which could be possible explanations for our result [27]. Additionally, subjective reporting has been shown to have low reliability [28]. Structured and contextual reporting using templates has been suggested to increase the clarity of radiology reports and make sure that important findings are assessed in a checklist fashion [29–31]. Existing visual rating scales might be a useful tool but their clinical use have been limited even though a few studies have shown they could improve the accuracy of radiological reporting [11, 14, 31–33]. Only a few studies have shown that the scales are applicable to NECT in clinical practice [9, 10, 14, 34]. Previous studies have shown an intra-rater agreement varying from moderate to strong for MTA and PCA [9, 11, 13, 17, 35, 36]. For WMC and GCA, intra-rater agreement have been substantial to strong in previous studies [9–11, 19]. Our results showed substantial to a strong intra-rater agreement for all pathologies but we used dichotomized data why the direct comparison with previous studies is more difficult. There is no clear consensus on what cut-offs should be used which we believe is a problem for the clinical application of the scales we used. This is true especially for the PCA and GCA scales which have a decreasing sensitivity in older patients [8, 12–18]. Both visual rating scales for MTA and PCA have been validated in comparison with volumetry and voxel-based morphometry and considered valuable tools in clinical practice [37, 38]. All of this considered, we do believe that visual rating scales might be clinically applicable to increase the accuracy of radiological reports but the question on what cut-offs to use still needs to be addressed and further studies on NECT are needed.

The second aim of our study was to compare the frequencies of reported findings among general radiologists and neuroradiologists. Neuroradiologists performed somewhat better than radiologists but MTA and PCA were underreported among neuroradiologists as well. Both MTA and PCA are important markers for AD and the combined presence of abnormal MTA and PCA increases both sensitivity and specificity for probable AD [15, 17, 39]. Global cortical atrophy is an unspecific finding which can be seen in many different conditions, including AD. White matter changes are associated with vascular dementia but they are also found in patients with AD suggesting a comorbidity [5, 8]. A combination of abnormal MTA and GCA also have a greater sensitivity and specificity for AD [15]. Evans’ index has been shown to increase with age but there is no significant difference between heathy elderly and patients with AD [22]. We believe that our results emphasize that MTA and PCA should be reported why we find the severe underreporting of MTA and PCA to be remarkable.

The third aim of our study was to compare the assessed pathologies with cognition. On CDT, patients with AD usually make conceptual errors and on the MMSE patients with AD usually score lower on items testing motor and working memory compared to patients with vascular dementia or Parkinson’s disease [40, 41]. Both pathological MTA and PCA have been shown to be associated with lower scores on MMSE [11, 15, 17]. Some degree of MTA can be present in healthy elderly, especially men, which reduces the sensitivity in older patients [15, 16, 42, 43]. We did not take gender into account and in our results, GCA, PCA, and EI/WLV showed an association with age but this did not sustain the Bonferroni correction. Other studies have shown that the level of education attenuates the effect of MTA on cognitive function, especially in older patients [43, 44]. The level of education showed no significant association with any cognitive domains in our study. White matter changes are associated with the decreased speed of processing, fluency, and working memory and especially periventricular WMC and also MTA has been shown to correlate with CDT performance [45, 46]. In our results, the association between pathological MTA and CDT and pathological MTA and ADAS were the only test to sustain the Bonferroni correction. This further emphasizes that MTA should be included in the radiology report.

Our study has some weaknesses. There is a selection bias of our study population. The included patients were retrospectively recruited from a regional research study with its predefined inclusion criteria and we do not know to what extent they are representative of the general population seeking primary care with SCD. We have no matched control groups meeting the MCI or AD criteria. The possibility of a regional bias cannot be excluded and we cannot assess to what extent our results are applicable to other regions or departments.

Due to the retrospective design of the study, there was a time gap between imaging and assessments of cognitive domains which could affect the validity of the NECT scans. None of the patients were diagnosed with dementia at baseline and since MTA, PCA, WMC, GCA, and EI/WLV represents long-term changes, we assumed that the validity of the scans would remain reasonable within a 12 months’ time gap. The retrospective design also resulted in patients being examined in different scanners and at different localities. All patients had been examined with local routine brain scan protocols and no dedicated dementia scan protocol. We believe that our dichotomization of data and a high degree of intra-rater reliability compensate for this and the validity of our ratings are reasonable within the scope of our study.

All visual ratings were performed by one person only, with high intra-rater reliability. Our rating scale cut-offs might differ from proposed cut-offs in the literature which could make comparisons with other studies difficult. The direct adaption of the scale used by Torisson et al. could also be questioned. This scale is an attempt to retrospectively quantify the interpretation of the radiology reports which could result in an interpretation bias of the original reports especially since this was also done by one rater. We have not studied the prospective impact of increased reporting and cannot draw any conclusions on whether patients with SCD actually would benefit from increased reporting of pathological findings or not.

The major strength of this study is that we have studied the potential use of visual rating scales in the everyday clinical assessment of NECT at both tertiary and secondary levels. Our results are in line with previous studies and reflect a need to improve the radiology reporting and increase the validity and reliability of structural neuroimaging in the primary diagnostic work-up in SCD [11, 14, 32]. We believe that proper training and the use of established visual rating scales in a contextual manner is one possible method to increase the correct reporting of pathological findings but this needs to be further investigated in a controlled clinical setting. We also believe it would be of interest to repeat this study and include data from other regions to investigate whether the underreporting of pathological findings suggestive of dementia is a general problem or not.

In conclusion, we propose that the implementation of visual rating scales in clinical practice should be emphasized to increase the yield of clinical CT exams in the diagnostic work-up in cognitive impairment. Special emphasis should be put on reporting MTA.
